# Targeting radiation‐induced upstream stimulatory factor‐1 by histone deacetylase inhibitors to reverse radioresistance in prostate cancer

**DOI:** 10.1002/cnr2.1553

**Published:** 2021-09-17

**Authors:** Seema Gupta, Mansoor M. Ahmed

**Affiliations:** ^1^ Department of Radiation Oncology University of Miami Miami Florida USA; ^2^ Present address: The Loop Immuno‐Oncology Laboratory, Lombardi Comprehensive Cancer Center Georgetown University Medical Center Washington, DC USA; ^3^ Present address: Radiation Research Program (RRP), Division of Cancer Treatment and Diagnosis (DCTD) National Cancer Institute/National Institutes of Health Rockville Maryland USA

**Keywords:** histone deacetylase inhibitors, PC‐3, radiation, upstream stimulatory factor‐1

## Abstract

**Background:**

Ionizing radiation (IR) is a standard modality for the management of solid tumors. Apart from its killing effects, IR can induce pro‐survival factors leading to radioresistance of cancer. Mechanistic understanding of radiation resistance is warranted to overcome the pro‐survival effects of IR.

**Aim:**

The aim of this study was to investigate the role of upstream stimulatory factor‐1 (USF‐1) in the induction of radioresistance in prostate cancer and its targeting by histone deacetylase (HDAC) inhibitors to reverse resistance.

**Methods and results:**

This study reports here that USF‐1 is a marker for radioresistance in PC‐3 cells. Using protein‐DNA array analysis, it was documented that DNA binding activity of USF‐1 was elevated following IR in PC‐3 cells. Novel HDAC inhibitors downregulated USF‐1 binding either alone or in combination with IR. A 5 Gy dose of IR induced the expression of target genes of USF‐1 (human telomerase reverse transcriptase [hTERT], IGF2R, CyclinB1, and Cdk1), however, HDAC inhibitors alone or in combination with IR reduced their expression as measured by real time RT PCR analysis. Furthermore, immunofluorescence analysis revealed that while USF‐1 localized primarily in the nucleus following IR, it localized in the cytoplasm when treated with HDAC inhibitors/combination. Maximum effects of modulation of USF‐1 expression (overexpression or suppression) were observed on hTERT activity as determined by dual‐luciferase reporter assay. To further confirm the role of USF‐1 in radioresistance, cell growth was analyzed using the real‐time cell electronic sensing (RT‐CES) system. This study found that USF‐1‐transfected cells proliferated faster than the vector‐transfected cells with or without treatments with HDAC inhibitors/IR/combination. Colony forming assay also confirmed that USF‐1 overexpression led to increased survival following IR. Importantly, colony‐forming assay demonstrated that HDAC inhibitors reversed the radioresistance in both PC‐3 and DU‐145 cells.

**Conclusion:**

These studies demonstrate that HDAC inhibitors reverse the radioresistance in prostate cancer through down‐modulation of USF‐1‐mediated transactivation of target genes involved in cell proliferation and cell cycle.

## 1 INTRODUCTION

Prostate cancer is the most frequently diagnosed non‐cutaneous cancer and the second leading cause of cancer‐related deaths among men in the United States.^1^ One of the most important problems in prostate cancer research is the need to identify a treatment for radiation resistant prostate cancer. Radiation resistance in prostate cancer may be implicated to induction of pro‐survival factors by ionizing radiation (IR) itself. These radiation‐induced pro‐survival factors may provide anti‐apoptotic signals to evade from cell killing effects of radiation. Pro‐survival signaling pathways such as STATs and NFκB have been extensively investigated and are previously shown to mediate the effects of IR.^2^, ^3^, ^4^, ^5^ However, role of upstream stimulatory factor (USF) in IR‐mediated effects has not been studied till now as per our knowledge, although, USFs are shown to be highly versatile stress responsive transcription factors.^6^

In mammals, USF proteins are encoded by two different genes, *Usf1* and *Usf2*, and these genes are ubiquitously expressed.^7^, ^8^ The cloning of USF‐1 and USF‐2 revealed that both proteins are members of the highly conserved family of bHLH‐LZ (basic‐Helix–Loop–Helix‐Leucine Zipper) proteins.^9^ It has been reported that USF transcription factors participate in distinct transcriptional processes, mediating recruitment of chromatin remodeling enzymes and interacting with co‐activators and members of the transcription pre‐initiation complex (reviewed in Reference [6]). USF proteins have been found to modulate gene transcription through their binding to cognate E‐box motifs leading to transcription stimulation. Further, interaction between USF‐1, and general, and cell‐specific transcription factors SP1, Pea3 and MTF1, respectively, for example, leads to cooperative transcriptional regulation. Furthermore, USF‐1 interacts directly with the transcriptional machinery of TATA‐plus and TATA‐less promoters. Finally, it has been shown that USF‐1 mediates recruitment of enzymes, such as PCAF that acetylates histones, and SET7/92 that methylates histone H3K4. These recruitments allow chromatin remodeling and opening, promoting DNA loading of the transcription machinery and transcription activation (reviewed in Reference [6]). Accordingly, USF‐1 interacts preferentially with highly acetylated histone H4 nucleosomal DNA.^10^ Results obtained from both cell lines and knockout mice indicate that USFs are key regulators of a wide number of gene regulation networks, including the stress and immune responses, cell cycle and proliferation, lipid and glucid metabolism, and in melanocytes. USF‐1 has been implicated as a key ultraviolet radiation (UV)‐activated regulator of genes associated with pigmentation (reviewed in Reference [6]).

Based on the above facts and since USF‐1 has been shown to upregulate the expression of several genes linked to cellular proliferation, it was of great interest to investigate whether inhibition of USF‐1 can result in radiosensitivity of prostate cancer cells. The location of the binding site within the promoter and the ability of USF to cooperate with other factors in regulating the gene expression may dictate the relative contribution of USF to any stress response. Since, these are strongly modulated by acetylation/deacetylation, histone deacetylase (HDAC) inhibitors might affect the functions of USF. HDACs have been shown to have fundamental importance in the initiation or progression of cancer and chromatin remodeling that is required for gene expression (similar to USF proteins),^11^, ^12^ therefore their targeting with inhibitors not only result in disruption of normal transcriptional regulation of specific genes through the relaxation of chromatin conformation but also has been used as a cancer therapy approach.

For most tumor cell lines derived from solid tumors, the primary effect of HDAC inhibition is that of cytostasis. Combination of HDAC inhibitors with radiation therefore has therapeutic advantages due to differential toxicity associated with each modality, potential for synergy due to physical interaction between HDAC inhibitors and chromatin architecture and differential expression of genes regulated by histone acetylation. The HDAC inhibitors—phenyl butyrate,^13^ sodium butyrate,^14^ TSA (trichostatin A),^15^, ^16^, ^17^ SAHA (suberoylanilide hydroxamic acid),^17^, ^18^, ^19^ M344,^17^ depsipeptide,^17^ and a benzamide MS‐275^20^ have shown tumor cell radiosensitivity in various cancer cell lines. Although, radiosensitizing effects of these inhibitors have been shown to be mediated through cell cycle arrest,^17^ inhibition of DNA synthesis and repair,^17^, ^20^, ^21^, ^22^, ^23^, ^24^ down‐regulation of anti‐apoptotic proteins, and upregulation of pro‐apoptotic proteins,^17^, ^23^, ^25^ the detailed understanding of the various signaling mechanisms of radiosensitization by these inhibitors is still lacking.

Therefore, this study was aimed to understand the regulatory roles of radiation‐induced USF‐1, its targeting by HDAC inhibitors, and to identify the novel mechanisms underlying the reversal of radioresistance, leading to radiosensitizing effects of these inhibitors in prostate cancer cells.

## 2 MATERIAL AND METHODS

### 2.1 Cell culture

Human prostate cancer line, PC‐3 (p53 null; androgen‐independent) and DU‐145 (p53 mutated; androgen‐independent) cells were obtained from American Type Culture Collection (ATCC). Cell lines were tested and authenticated by RADIL (now IDEXX BioAnalytics, University of Missouri‐Columbia), using short tandem repeat markers. The alleles for nine different short tandem repeat markers were determined for each sample, and the results were compared with the genetic profiles reported by the ATCC for each cell line. The genetic profiles for the samples were consistent with the genetic profiles reported by ATCC for each cell line and no cross‐contamination with other species was observed. Cells were cultured in RPMI supplemented with 10% fetal bovine serum and 1% penicillin streptomycin at 37°C and 5% CO_2_.

### 2.2 HDAC inhibitors

Based on the X‐ray crystallographic structure of HDAC enzyme, Zn^2+^‐chelating, motif‐tethered, short chain fatty acids were developed as novel class of HDAC inhibitors.^26^ Two of these inhibitors, VAD‐18 (V18) and VAD‐20 (V20) having phenylacetic acid and butyric acid, respectively as the lead compound with another novel compound S‐HDAC‐42 (S‐42; AR‐42)^27^ and SAHA formulated in dimethylsulphoxide (DMSO) at a stock concentration of 40 mM were used in this study. Since, these novel inhibitors have aromatic chain (rather than aliphatic chain present in most other inhibitors) as the linker between the lead compound and Zn^2+^ chelating hydroxamic acid, there is more strong interaction between the hydrophobic pocket of the active site of the enzyme and the inhibitor thus increasing the potency of inhibition.^26^, ^27^

### 2.3 Stable and transient transfections

To confirm the role of USF‐1 in IR‐induced signaling, stable transfectants of PC‐3 cells were generated by either over‐expressing USF‐1 with USF‐1 flag‐tagged plasmid or vector (kindly provided by Dr. Janknecht, Rochester, MN) or suppressing USF‐1 expression using SureSilencing ShRNA Plasmid for human USF‐1 or negative control for USF‐1 (SuperArray Bioscience Corporation) using effectene transfection reagent (Qiagen; Cat no.: 301427). Stable transfectants were selected with geneticin (GIBCO; 1000 μg/ml). Expression of USF‐1 in these cells was confirmed by real time reverse transcriptase‐polymerase chain reaction (RT PCR) and western blot analysis.

PC‐3 and PC‐3‐USF‐1 cells were transiently transfected with pRLTK (5 ng) and wild‐type (wt) or mutant hTERT(0.25 μg) (luciferase reporter corresponding to either the core hTERT promoter [−233/+438] or the mutated downstream [+44 to +49] and upstream [−165 to −160] E‐boxes [the binding sites for USF‐1]) (kindly provided by Dr. Janknecht, Rochester, MN)^28^ using effectene transfection reagent according to manufacturer's directions (Qiagen). PC‐3 cells were also transfected with USF‐1 expression plasmid (0.75 μg).

### 2.4 Cell treatments

A 100 kV industrial X‐ray machine (Phillips) was used to irradiate the cells at room temperature. The dose rate with a 2 mm Al plus 1 mm Be filter was ~2.64 Gy/min at a focus surface distance of 10.5 cm.

Cells were either left untreated or exposed to 1–6 Gy dose of radiation or to different concentrations of HDAC inhibitors. For combined treatments, the cells were treated with IC_50_ concentrations of the drugs and were exposed to radiation (2 Gy/5 Gy) immediately without changing the medium.

### 2.5 Colony forming assay

Clonogenic survival assays were performed for each treatment group as described previously.^29^, ^30^ *D*_0_ values were calculated using single hit multiple target model. Radiation enhancement ratios were calculated as described previously.^30^

### 2.6 Western blot analysis

Total protein was extracted from cells following transfection using Laemmli buffer and subjected to western blot analysis as described earlier.^29^, ^30^ After electrophoresis, the separated proteins were transferred to PVDF membrane using wet method. The membranes were incubated with rabbit polyclonal antibody to USF‐1 (SC‐229; Santa Cruz Biotechnology). The bound antigen–antibody complex was detected by HRP conjugated secondary antibody (Santa Cruz Biotechnology) and the electrochemiluminescence plus western blot detection system (GE Healthcare UK Limited). The same membrane was used for β‐actin levels detected by anti‐β‐actin antibody (Sigma Chemical Company) as an internal loading control.

### 2.7 Protein‐DNA array

24 h after various treatments, Protein‐DNA Array (TranSignal TF Protein Array) (Panomics) analysis was carried out according to the manufacturer's instructions in PC‐3 cells to identify the role of 54 transcription factors.

### 2.8 Real time RT PCR

Total RNA was extracted from the cells 24 h after various treatments using TRIzol reagent (Life Technologies, Inc.). One μg of total RNA was reverse transcribed into cDNA (SuperArray Bioscience Corporation; RT^2^ first strand kit; Cat. No. C‐03) using polymerase chain reaction (PCR). Real time PCR was then performed using primers (USF‐1; hTERT; IGF2R; CDK1; CYCLIN B1; β actin), reagents (Taqman Universal master mix) and instrument (AB7300) from Applied Biosystems, Foster City, CA, USA. Data were analyzed using Applied Biosystems' and SA Biosciences' softwares. Gene expression was normalized with the beta‐actin gene expression.

### 2.9 Dual‐luciferase reporter assay

24 h post‐transfection, cells were either left untreated or irradiated (5 Gy). 24 h after treatment, the cells were lysed in reporter lysis buffer and subjected to 3 freeze–thaw cycles. The activity of luciferase was measured using a Dual‐Luciferase Reporter assay (Promega) using single tube Luminometer, TD20/20 (Turner Biosystems) and expressed as a ratio of hTERT or mt hTERT/pRL‐TK for normalization.

### 2.10 Immunofluorescence assay

PC‐3 cells were cultured on Lab‐Tek chamber slides (Nunc Inc.) and 24 h after various treatments were fixed in buffered formalin. Non‐specific sites were blocked with 3% bovine serum albumin (BSA) in phosphate buffered saline (PBS) (pH 7.4) with 0.25% Tween 20 for 30 min. Slides were then incubated overnight at 4°C in primary rabbit polyclonal antibody to USF‐1 (SC‐229; Santa Cruz Biotechnology) that was diluted to 1:100 with the blocking buffer. After washes in PBS (pH 7.4) with 0.25% Tween 20 (3 times each for 15 min), the cells were exposed to secondary antibody, FITC‐conjugated anti‐rabbit IgG that was diluted to 1:1000 in blocking buffer. After three washes in PBS (pH 7.4) with 0.25% Tween 20 (3 times each for 15 min) and one wash in PBS for 15 min, the slides were mounted with aqueous mounting media using antifade and DAPI (4′,6‐diamidino‐2‐phenylindole) (VectaShield, Vector) and visualized using triple band pass filter in Nikon epifluorescence microscope.

### 2.11 Cell growth assay

Stable transfectants of PC‐3 with vector or USF‐1 were grown on the surfaces of microelectronic sensors, which are comprised of circle‐on‐line electrode arrays and are integrated into the bottom surfaces of the 16‐well plate (ACEA Biosciences). Changes in cell number were monitored and quantified by detecting sensor electrical impedance. Cells were treated with V18, V20, or S‐42 alone or in combination with IR (5 Gy) in duplicates. The dynamic response of the cells to the treatments was continuously monitored by real time‐cell electronic sensing (RT‐CES) system (ACEA Biosciences). Cell number was normalized to the cell number at the time of treatment.

### 2.12 Statistical methods

All the experiments were either performed in duplicates or triplicates as mentioned in the respective figure legends. All summary statistics (average values, standard error of mean [SEM], significant differences between groups) were calculated using GraphPad Prism v.7.0. Statistical significance between groups was determined by unpaired, one‐tailed Student's *t*‐test or one‐way analysis of variance (ANOVA) (*p* ≤ .05 was considered statistically significant).

## 3 RESULTS

### 3.1 Identification of transcription factors modulated by histone deacetylase inhibitors in combination with radiation

Binding activity of 54 transcription factors was analyzed in PC‐3 cells following 2 Gy irradiation using TranSignal protein/DNA array. It was observed that the binding activity of the most of the factors involved in the transcription initiation machinery (C/EBP, p65, CREB and AP‐1) (reviewed in Reference [31]) or its activators like NFATc,^32^ STATs, NFκB, and USF‐1 were either upregulated or induced in PC‐3 cells following 2 Gy irradiation compared to untreated group (Table 1).

**TABLE 1:** TranSignal protein‐DNA array was carried out according to the manufacturer's instructions in PC‐3 cells 24 h after 2 Gy exposure. Binding activity of various transcription factors is presented compared to the controls

Genes	2 Gy
Upregulated^a^	CBF, AP‐1 (new), MEF‐2, NFκB, Stat1, Stat3, Stat5, USF‐1, HSE
Induced^b^	AP‐1, C/EBP, CREB, E2F1, ERE, FAST‐1, GAS/ISRE, GATA, GRE, HNF‐4, Myc‐Max, NFATc, NF‐E1, Oct‐1, Sp1

^a^ Increased binding activity in the treated group compared to the control group.

^b^ Binding activity was absent in the control group but was present in the irradiated group.

**FIGURE 1:**
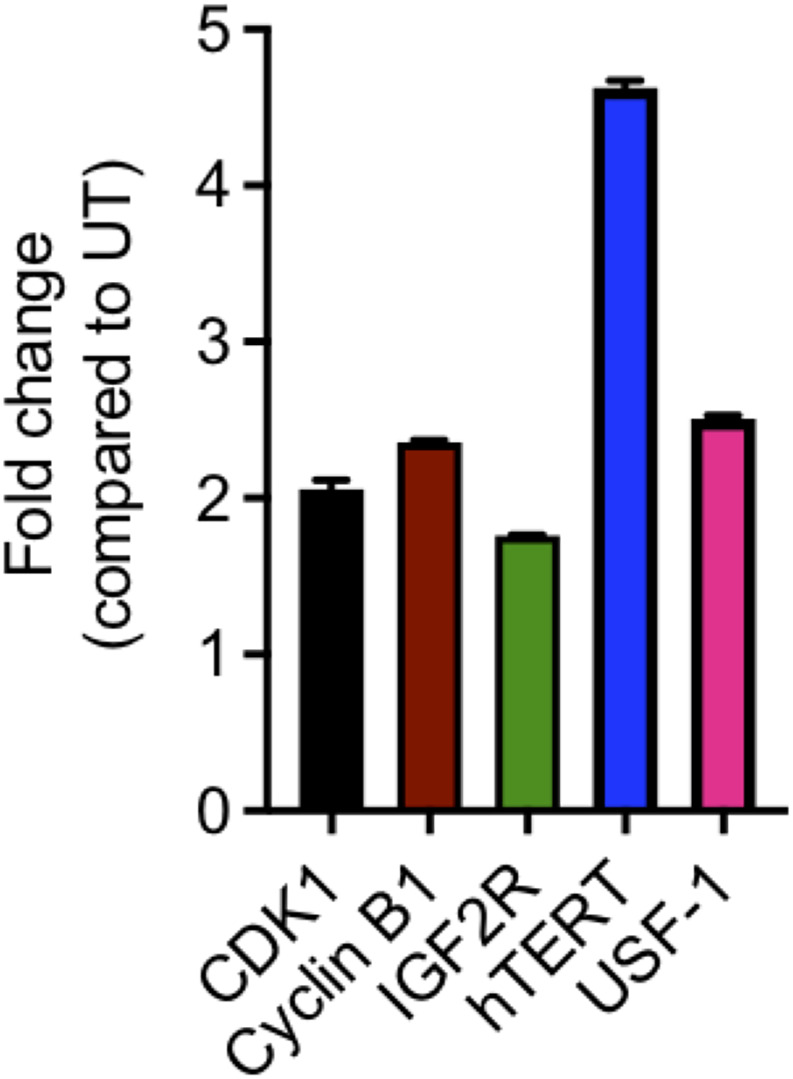
Expression of USF‐1 and its targets after ionizing radiation. In PC‐3 cells, 24 h after radiation (5 Gy), RNA was isolated and cDNA was prepared using PCR with 1 μg of RNA. Real time PCR was then performed using primers for CDK1, CYCLINB1, IGF2R, hTERT, and USF‐1 in duplicates. Results are presented after normalization with untreated group and using β‐actin as the endogenous control. Expression of USF‐1 and its targets was significantly upregulated compared to the untreated group after irradiation. The data shown are the average of two independent experiments. The error bars show mean ± SEM

Since, role of USF‐1 in IR‐mediated effects has not been studied till now as per our knowledge, we studied the expression of its target proteins that are associated with carcinogenesis or proliferation and cell cycle and have relevance in radiation response such as hTERT,^28^ IGF2R,^33^ Cyclin B1,^34^ and Cdk1^35^ by real time RT PCR in PC‐3 cells. An induction in gene expression was observed following irradiation of cells with 5 Gy dose for all the four genes (Figure 1). It has been reported that USF‐1 expression does not change in response to the stress, suggesting that post‐translational modifications and interaction with other proteins are important for USF‐1's regulatory functions.^28^, ^36^ However, in PC‐3 cells, a significant increase in expression of USF‐1 was observed following IR exposure (Figure 1), indicating that IR‐induced stress signaling may be different than other type of stress like UV.

### 3.2 Regulation of human telomerase reverse transcriptase expression by USF‐1 and increase in survival of USF‐1‐transfected PC‐3 cells following irradiation

**FIGURE 2:**
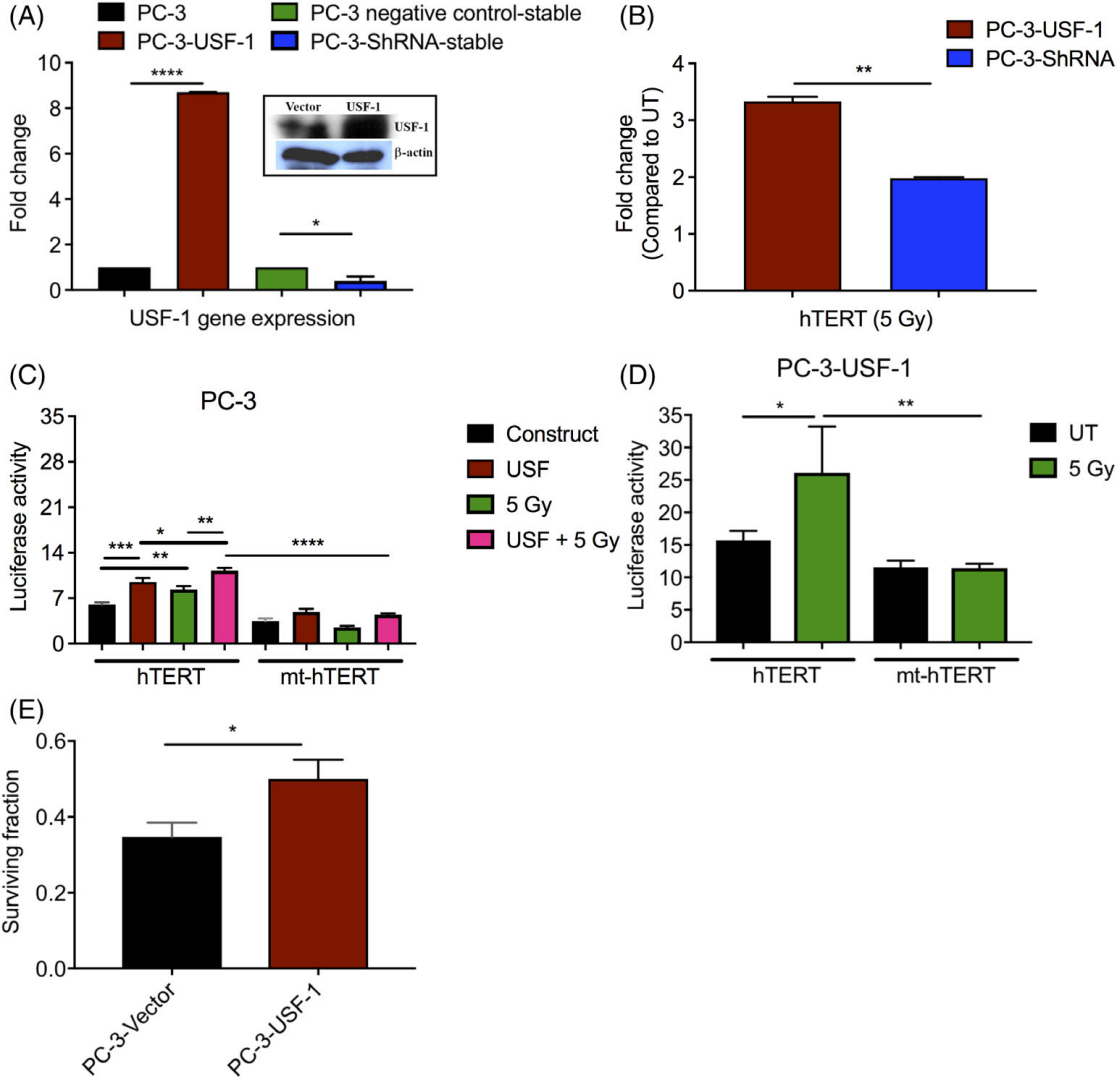
Role of USF‐1 in radioresistance. (A) PC‐3 cells were transfected with vector or USF‐1 flag‐tagged plasmids or ShRNA or negative control for USF‐1 using effectene transfection kit. Stable transfectants were selected with geneticin (1000 μg/ml). Expression of USF‐1 in these cells was confirmed by real time RT PCR. The data shown are the average of two independent experiments. The error bars show mean ± SEM; *p ≤ 0.05; ****p ≤ 0.0001. Inset shows the western blot for USF‐1 protein in USF‐1 plasmid‐transfected PC‐3 whole cell lysates using anti‐USF‐1 antibody. β‐actin was used as the endogenous control. (B) 24 h after 5 Gy irradiation of PC‐3‐USF‐1 and PC‐3‐ShRNA cells, RNA was isolated and cDNA was prepared using PCR with 1 μg of RNA. Real time PCR was then performed using hTERT primers in duplicates. Results are presented after normalization with untreated group and using β‐actin as the endogenous control. The data shown are the average of two independent experiments. The error bars show mean ± SEM; **p ≤ 0.01. (C) PC‐3 and (D) PC‐3‐USF‐1 cells were transiently transfected with pRLTK (5 ng) and wild‐type or mutant hTERT (0.25 μg). PC‐3 cells were also transfected with USF‐1 (0.75 μg). 24 h after transfection, cells were either left untreated or irradiated (5 Gy). 24 h after, luciferase activity was measured using dual‐luciferase reporter assay system kit in triplicates. The data shown are the average of three independent experiments. The error bars show mean ± SEM; *p ≤ 0.05; **p ≤ 0.01; ***p ≤ 0.001; ****p ≤ 0.0001. (E) Effects of 2 Gy irradiation on survival of stable transfectants of PC‐3‐Vector or PC‐3‐USF‐1 cells by colony forming assay. Cells were counted and plated in quadruplicates. Once the cells were attached, they were exposed to radiation and incubated at 37°C for colony formation. The colonies were stained and colonies having more than 50 cells were counted. The data shown are the average of two independent experiments. The error bars show mean ± SEM; *p ≤ 0.05

To confirm the role of USF‐1 in IR‐induced signaling, we generated stable transfectants of PC‐3 cells either overexpressing USF‐1 using USF‐1 plasmid (PC‐3‐USF‐1) or suppressed USF‐1 expression using ShRNA for USF‐1 (PC‐3‐ShRNA). Expression of USF‐1 was confirmed by real time RT PCR and western analysis (Figure 2A).

Expression of hTERT was analyzed in PC‐3‐USF‐1 and PC‐3‐ShRNA cells by real time RT PCR following 5 Gy irradiation. Increased expression of hTERT was observed in PC‐3‐USF‐1 cells compared to PC‐3‐ShRNA cells (Figure 2B), indicating that USF‐1 overexpression is responsible for upregulation of hTERT. In cells transfected with ShRNA to USF‐1, hTERT expression was still more than untreated (UT), which could be due to the incomplete suppression of USF‐1 as shown in Figure 2A.

To further confirm that this increase in hTERT expression is regulated by USF‐1, luciferase reporter assays were performed in PC‐3 and PC‐3‐USF‐1 cells after transiently transfecting cells with the luciferase reporter corresponding to either the core hTERT promoter (−233/+438) or the mutated downstream (+44 to +49) and upstream (−165 to −160) E‐boxes (the binding sites for USF‐1)^28^ and USF‐1 expression plasmid. Following 5 Gy irradiation of cells, the increase in hTERT reporter activity was observed only in wt‐hTERT‐transfected cells both in PC‐3 and PC‐3‐USF‐1 cells (Figure 2C,D). Mutation of the E‐boxes significantly abrogated promoter responsiveness to USF‐1 (Figure 2C,D), indicating that hTERT expression is regulated by USF‐1 following IR.

In line with the above data, PC‐3 cells stably transfected with USF‐1 showed increased survival after 2 Gy irradiation compared to cells transfected with vector by colony forming assay (Figure 2E).

### 3.3 Radiosensitization by histone deacetylase inhibitors

Based on the role of USF‐1 in radioresistance that was confirmed by USF‐1 gain of function studies in PC‐3 cells using real time RT PCR, reporter assays and colony forming assays, and since USF‐1 has been shown to upregulate the expression of several genes linked to cellular proliferation, it was of great interest to investigate whether inhibition of USF‐1 can result in radiosensitivity of prostate cancer cells, in turn reversing radioresistance. It may be possible to inhibit the functions of radiation‐induced pro‐survival factors like USF‐1 and enhance radiation‐induced apoptosis by the use of several drugs. Since the transcriptional activity of USF‐1 may be modulated by acetylation/deacetylation, we investigated the radiosensitizing effects of the novel HDAC inhibitors in PC‐3 and DU‐145 cells either alone or in combination with ionizing radiation treatment by colony forming assays.

**FIGURE 3:**
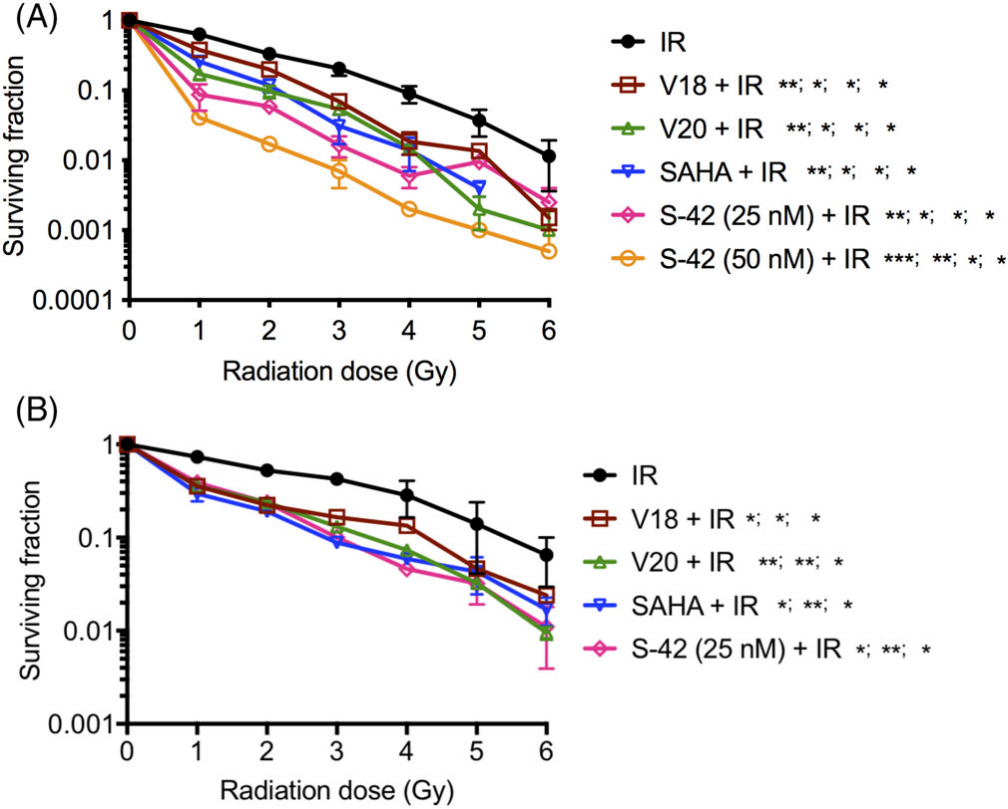
Radiosensitization by HDAC inhibitors. Effects of (A) V18 (0.5 μM), V20 (7.5 μM), SAHA (0.2 μM), and S‐42 (25 and 50 nM) in combination with radiation (1–6 Gy) on surviving fraction of PC‐3 cells (*p* values are shown for 1–4 Gy; at 5 and 6 Gy significant differences were not found between IR and HDAC inhibitors + IR groups) and (B) V18 (0.4 μM), V20 (7.0 μM), SAHA (0.25 μM) and S‐42 (25 nM) in combination with radiation (1–6 Gy) on surviving fraction of DU‐145 cells (*p* values are shown for 1–3 Gy; at 4–6 Gy significant differences were not found between IR and HDAC inhibitors + IR groups) studied by colony forming assays. As described for Figure 2E, cells were plated in quadruplicates for each treatment. The data shown are the average of two independent experiments. The error bars show mean ± SEM; *p ≤ 0.05; **p ≤ 0.01; ***p ≤ 0.001

Cells were treated with various concentrations of V18, V20, S‐42, and SAHA to find the IC_50_ concentration for each drug. The decrease in surviving fraction with increasing concentration was observed with all the drugs in both PC‐3 and DU‐145 cell lines (Figure SS1A,B). S‐42 was most cytotoxic followed by SAHA, V18, and V20 (corresponding IC_50_ concentrations in PC‐3 and DU145 cells are given in Tables SS1 and S2). The effects of HDAC inhibitors in combination with radiation (1–6 Gy) on survival of PC‐3 and DU‐145 cells are presented in Figure 3A,B respectively. Significant radiosensitizing effects with all the inhibitors at IC_50_ concentrations compared to IR alone were observed in both the cell lines (Tables SS1 and S2). S‐42 was able to reverse the radioresistance much more effectively than any of the other HDAC inhibitors, V18, V20, or SAHA in PC‐3 cells (Figure 3A; Table SS1). However, in DU‐145 cells, all the HDAC inhibitors demonstrated similar effects on the survival of cells (Figure 3B; Table S2).

### 3.4 Reduction in the expression of radiation‐induced targets of USF‐1 by histone deacetylase inhibitors

Next, effects of these novel inhibitors alone or in combination with radiation on binding activity of USF‐1 were analyzed by protein‐DNA array in PC‐3 cells. The array results are tabulated for the most important factors that showed differences in expression compared to untreated group in Table 2. Most of the STATs and USF‐1 were down‐regulated following treatment with HDAC inhibitors alone or in combination with radiation (Table 2).

**TABLE 2:** TranSignal protein‐DNA array was carried out according to the manufacturer's instructions in PC‐3 cells 24 h after V18 alone or V18 + 2 Gy treatment. Binding activity of various transcription factors is presented compared to the controls

Genes	V18	V18 + 2 Gy
Downregulated^a^	Stat1, Stat3, Stat5, TR (DR‐4), USF‐1, VDR (DR‐3)	TR (DR‐4), USF‐1
Repressed^b^	HSE	‐

^**a**^ Reduced binding activity in the treated group compared to the control group.

^b^ Transactivation function was absent in the treated group but was present in the control group.V18, VAD‐18.

**FIGURE 4:**
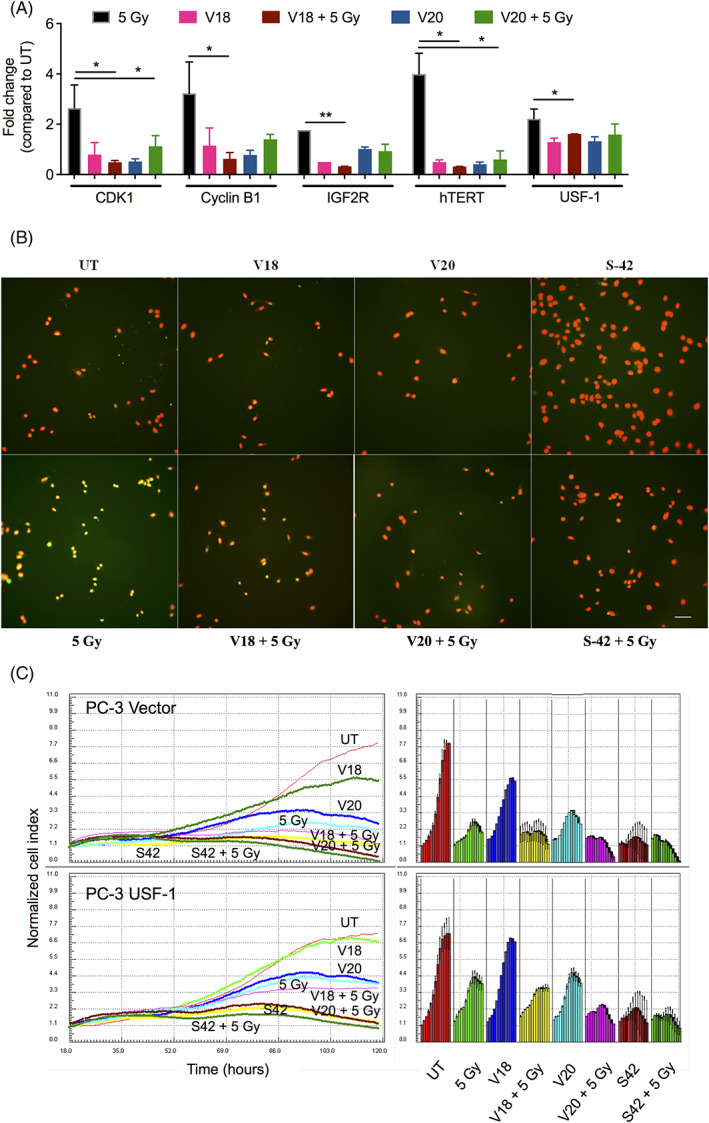
Reduction of USF‐1 function and USF‐1–mediated survival by HDAC inhibitors. (A) In PC‐3 cells, 24 h after various treatments with HDAC inhibitors alone or IR radiation alone (5 Gy) or their combination, RNA was isolated and cDNA was prepared using PCR with 1 μg of RNA. Real time PCR was then performed using primers for CDK1, CYCLINB1, IGF2R, hTERT, and USF‐1 in duplicates. Results are presented after normalization with untreated group and using β‐Actin as the endogenous control. The data shown are the average of two independent experiments. The error bars show mean ± SEM; *p ≤ 0.05; **p ≤ 0.01. (B) 24 h after various treatments, PC‐3 cells were fixed in buffered formalin. Slides were then incubated overnight at 4°C in primary rabbit polyclonal antibody to USF‐1 and then cells were exposed to secondary antibody, FITC‐conjugated anti‐rabbit IgG. The slides were mounted with aqueous mounting media using antifade and DAPI and visualized using triple band pass filter in Nikon epifluorescence microscope. The DAPI and FITC images were merged to identify the subcellular localization of USF‐1. Images were acquired at a magnification of 20X. Representative data from one of the two experiments is shown. Scale bars: 10 μm. (C) Stable transfectants of PC‐3 with vector or USF‐1 were grown on the surfaces of microelectronic sensors, which are comprised of circle‐on‐line electrode arrays and are integrated into the bottom surfaces of the 16‐well plate. Changes in cell number were monitored and quantified by detecting sensor electrical impedance. Cells were treated with V18, V20, or S‐42 alone or in combination with IR (5 Gy) in duplicates. The dynamic response of the cells to the treatments was continuously monitored by RT‐CES system. Cell number was normalized to the cell number at the time of treatment

To confirm that expression of USF‐1 targets is also abrogated, we performed real time RT PCR. As expected, the HDAC inhibitors were able to reduce the expression of radiation‐induced targets of USF‐1 either alone or in combination with IR (Figure 4A).

### 3.5 Cytoplasmic localization of USF‐1 following treatment with histone deacetylase inhibitors

It has been reported that post‐translational modifications like phosphorylation may regulate the function of USF‐1.^36^, ^37^ USF‐1 being a transcription factor localizes in the nucleus to mediate its functions. Therefore, we investigated the localization of USF‐1 following IR by immunofluorescence and observed that USF‐1 was localized in the nucleus following 5 Gy irradiation compared to UT group (Figure 4B). Incubation of PC‐3 cells with HDAC inhibitors alone or in combination of radiation resulted in cytoplasmic localization of USF‐1 compared to its nuclear localization when the cells were treated with radiation alone (Figure 4B). These results suggest that pro‐survival and proliferation effects mediated by USF‐1 are inhibited in the presence of HDAC inhibitors.

### 3.6 Increased cell growth of PC‐3‐USF‐1 cells compared to PC‐3 cells

Further, to confirm the role of USF‐1 in radioresistance, effects of HDAC inhibitors alone or in combination with IR on cell proliferation were studied in PC‐3‐vector or PC‐3‐USF‐1 cells by RT‐CES system. Increased proliferation of USF‐1 transfected PC‐3 cells treated with either HDAC inhibitors alone, IR alone or in combination was observed (Figure 4C). However, HDAC inhibitors were able to reduce the proliferation of cells compared to IR even in the cells overexpressing USF‐1. Results from RT‐CES (Figure 4C) and colony forming assay (Figure 2E) using stably transfected PC‐3 cells with vector or USF‐1 suggest that reversal of radioresistance by HDAC inhibitors may be mediated through inhibition of USF‐1.

## 4 DISCUSSION

In the present study, it is demonstrated for the first time that the IR can induce transcription factor, USF‐1 and the expression and transcriptional activity of its targets involved in cell proliferation, and cell cycle are enhanced in cells overexpressing USF‐1. This suggests that USF‐1 can contribute towards the radioresistance in prostate cancer as demonstrated by the overexpression studies. When USF‐1 was overexpressed in PC‐3 cells, an increased growth and survival was observed following IR.

USF transcription factors may have a complex role in cancers as both their carcinogenic and anti‐carcinogenic effects in different types of cancer have been reported.^38^, ^39^, ^40^ While, in prostate cancer cells and breast cancer cell lines, it has been shown that loss of USF transcriptional activity is associated with carcinogenesis, in lung cancer it was demonstrated that USF‐2 represents an early marker for the development of bronchial dysplasia and non‐adenocarcinoma,^38^, ^39^, ^40^ thus suggesting a complex role for these transcription factors. The location of the binding site within the promoter and the ability of USFs to cooperate with other factors in regulating the gene expression may dictate the relative contribution of USFs to any stress response. Since, these are strongly modulated by acetylation/deacetylation, it is hypothesized that HDAC inhibitors might affect the functions of USFs.

HDAC inhibitors have been shown as radiosensitizers in some colon, glioma, squamous cell carcinoma, and prostate cancer cell lines.^13^, ^14^, ^15^, ^16^, ^17^, ^20^ However, the mechanisms by which they act as radiosensitizers have not been well elucidated. Further, HDAC inhibitors have advanced to clinical trials but there has been no focus on utilizing their radiosensitizing effects.^27^ Present results show that novel HDAC inhibitors, V18, V20, and S‐42 are potent radiosensitizers of prostate cancer cells. These inhibitors have shown hyperacetylation of histones H‐3 and H‐4 in DU‐145 prostate cancer cells in a dose dependent manner and at much lower doses required by the parent molecule indicating that they are potent HDAC inhibitors.^26^

Many factors, including specific DNA sequences, histones, non‐histone chromosomal proteins, transcriptional activators/repressors, and the transcription machinery are all necessary for the establishment of an active transcription complex.^41^ Condensation of eukaryotic DNA in chromatin suppresses gene activity through the coiling of DNA on the surface of the nucleosome core and the folding of nucleosome assemblies, thus decreasing the accessibility to the transcriptional apparatus.^42^ HDACs not only cause the inhibition of gene transcription, but also directly affect the nuclear activity of transcription factors such as NFκB.^43^ It is highly probable that nuclear activation of USF‐1 similar to NFκB is dependent upon the activity of HDACs, providing an acetylation balance dependent mechanism for the regulation of USF‐1‐mediated transcription. In addition, the interaction of USF‐1 with other cooperative factors may be influenced in the presence of HDAC inhibitors as well as its direct binding to chromatin may be modulated, all affecting its transcriptional activity. In fact, USFs have been shown to influence the transcription of several genes, regulating cellular growth and suppression, lipid and glucose metabolism and so forth, through its binding to the E‐boxes present in their promoters.^44^ USF‐1/2 bind to the two E‐boxes in the hTERT promoter as a heterodimer, stimulating the transcription of hTERT.^28^ Similarly, *igf2r*, *cyclin B1, cdk1* and others have been demonstrated as USF‐specific targets where USF stimulates their transcription via E‐box/es binding.^33^, ^34^, ^44^ In addition, COX‐2, a known mediator of tumor resistance to radiotherapy,^45^ can be one of the other potential signaling molecules modulated by USF. In fact, USF1/2 is reported to bind to COX‐2 E‐box in gastroenteropancreatic neuroendocrine tumors.^46^ USF is also shown to regulate the transcription of COX‐2 in mouse skin carcinoma cells.^47^

Indeed, HDAC inhibitors could reduce the expression of radiation‐induced USF‐1 and its targets involved in cell proliferation and cell cycle (Figure 4A). Immunofluorescence results clearly demonstrated that USF‐1 is mostly localized in the cytoplasm when PC‐3 cells were treated with these novel HDAC inhibitors alone or in combination with radiation, in contrast to radiation treatment where the localization was nuclear (Figure 4B). It has been reported that phosphorylated USF‐1 is responsible for transcriptional regulation following various kinds of stress.^37^ However, in the second step if this phosphorylated form is acetylated dependent on the extent of stress, the transcriptional regulation of USF‐1 is lost.^37^ Indeed, enhanced acetylation of this phosphorylated site by HDAC inhibitors may lead to loss of transcriptional regulation by USF‐1 thereby reducing the expression of its target genes leading to radiosensitization. However, in contrast to the results presented here the sub‐cellular localization of the acetylated‐phosphorylated USF‐1 remained nuclear in melanoma cells.^37^ This could be due to the different kind of stress (radiation vs. hydrogen peroxide [H_2_O_2_]/methyl methane sulphonate [MMS]), different cellular context (prostate cancer vs. melanoma) or different kind of deacetylation inhibition (novel HDAC inhibitors vs. site directed mutagenesis/TSA A). The epigenetic analysis of PC‐3 cells after IR in future may provide newer insights into the USF‐mediated transcription and its effects on radioresistance. Furthermore, pre‐clinical studies in animal tumor models and tumor types other than prostate cancer will further enhance our understanding of role of USF‐1 as a mediator of radioresistance.

The identification of USF‐1 as a putative target for reversing the radioresistance by HDAC inhibitors has opened a new paradigm for prostate cancer therapy. In addition, this study shows that novel HDAC inhibitors, V18, V20, and S‐42 are potent radiosensitizers of PC‐3 cells. Future studies that will further help in understanding the role of USF‐1 in IR and HDAC inhibitors‐induced signaling in other prostate cancer cells will lead to development of better drugs and treatment strategies for cancer therapy.

## ETHICAL STATEMENT

Not applicable

## CONFLICT OF INTEREST

The authors declare no conflict of interest.

## AUTHOR CONTRIBUTIONS

Seema Gupta and Mansoor M. Ahmed conceived the study and designed the experiments. Seema Gupta performed the experiments. Seema Gupta and Mansoor M. Ahmed analyzed the data. Seema Gupta wrote the original draft, and Seema Gupta and Mansoor M. Ahmed reviewed and edited the manuscript.

## Supporting information


**Figure S1** Effects of various concentrations of V18, V20, SAHA, and S‐42 on surviving fraction of (A) PC‐3 cells and (B) DU‐145 cells studied by colony forming assays. As described for Figure 2E, cells were plated in quadruplicates for each treatment. The data shown are the average of two independent experiments. The error bars show mean ± SEM.

**Table S1** Inactivation estimates of various HDAC inhibitors in PC‐3 cells with or without radiation and radiation enhancement ratios.
**Table S2:** Inactivation estimates of various HDAC inhibitors in DU‐145 cells with or without radiation and radiation enhancement ratios.

## Data Availability

Data related to main and supplementary figures are included in this published article. All other relevant data are available from the corresponding author upon reasonable request.
