# Supplementary figures and images for: Targeting radiation‐induced upstream stimulatory factor‐1 by histone deacetylase inhibitors to reverse radioresistance in prostate cancer
Source: Cancer Rep (Hoboken). 2021 Sep 17;5(12):e1553. doi: 10.1002/cnr2.1553 (PMC9780427; doi:10.1002/cnr2.1553)

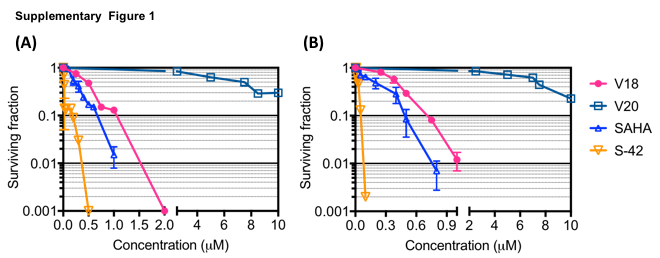

Supplement: Supplementary file 1 — Figure S1 Effects of various concentrations of V18, V20, SAHA, and S‐42 on surviving fraction of (A) PC‐3 cells and (B) DU‐145 cells studied by colony forming assays. As described for Figure 2E, cells were plated in quadruplicates for each treatment. The data shown are the average of two independent experiments. The error bars show mean ± SEM. [file CNR2-5-e1553-s001.tiff]
