# Supplementary material for: Targeting radiation‐induced upstream stimulatory factor‐1 by histone deacetylase inhibitors to reverse radioresistance in prostate cancer
Source: Cancer Rep (Hoboken). 2021 Sep 17;5(12):e1553. doi: 10.1002/cnr2.1553 (PMC9780427; doi:10.1002/cnr2.1553)
Supplement: Supplementary file 2 — Table S1 Inactivation estimates of various HDAC inhibitors in PC‐3 cells with or without radiation and radiation enhancement ratios. Table S2: Inactivation estimates of various HDAC inhibitors in DU‐145 cells with or without radiation and radiation enhancement ratios. [file CNR2-5-e1553-s002.pdf]

**Supplementary Table 1:** Inactivation estimates of various HDAC inhibitors in PC-3 cells with or without radiation and radiation enhancement ratios.

| Treatment | Inactivation estimates | | | Radiation enhancement ratios |
|---|---|---|---|---|
| | $IC_{50}$ | $SF_2$ | $D_0$ (cGy) | |
| Radiation (IR) | - | 0.33 ± 0.031 | 148.8 | - |
| V18 | 0.5 μM | - | - | - |
| V20 | 7.5 μM | - | - | - |
| V18 + IR | - | 0.2 ± 0.013 | 145 | 1.65 |
| V20 + IR | - | 0.1 ± 0.02 | 122 | 3.3 |
| S-42 | 20 nM | - | - | - |
| S-42 + IR | - | 0.0585 ± 0.0015 | 42 | 5.64 |
| SAHA | 0.2 μM | - | - | - |
| SAHA + IR | - | 0.118 ± 0.025 | 79 | 2.8 |

**Supplementary Table 2:** Inactivation estimates of various HDAC inhibitors in DU-145 cells with or without radiation and radiation enhancement ratios.

| Treatment | Inactivation estimates | | | Radiation enhancement ratios |
|---|---|---|---|---|
| | $IC_{50}$ | $SF_2$ | $D_0$ (cGy) | |
| Radiation (IR) | - | 0.52 ± 0.025 | 275 | - |
| V18 | 0.4 μM | - | - | - |
| V20 | 7.0 μM | - | - | - |
| V18 + IR | - | 0.22 ± 0.038 | 128 | 2.36 |
| V20 + IR | - | 0.24 ± 0.0045 | | 2.17 |
| S-42 | 25 nM | - | - | - |
| S-42 + IR | - | 0.24 ± 0.0045 | 121 | 2.17 |
| SAHA | 0.25 μM | - | - | - |
| SAHA + IR | - | 0.19 ± 0.0045 | 99.5 | 2.74 |
